# Influence of Metabolite Extraction Methods on ^1^H-NMR-Based Metabolomic Profiling of Enteropathogenic *Yersinia*

**DOI:** 10.3390/mps1040045

**Published:** 2018-11-20

**Authors:** Brandon R. Gines, Willard E. Collier, Mohamed A. Abdalla, Teshome Yehualaeshet

**Affiliations:** 1 Department of Pathobiology, Tuskegee University, Tuskegee, AL 36088, USA; bgines5885@tuskegee.edu; 2 Department of Chemistry, Tuskegee University, Tuskegee, AL 36088, USA; wcollier@tuskegee.edu (W.E.C.); abdalla@tuskegee.edu (M.A.A.)

**Keywords:** nuclear magnetic resonance, metabolomics, metabolite extraction methods, *Yersinia*

## Abstract

Metabolite extraction is one of the critical steps in microbial metabolome analysis. It affects both the observed metabolite content and biological interpretation of the data. Several methods exist for metabolite extraction of microbes, but the literature is not consistent regarding the sample model, adequacy, and performance of each method. In this study, an optimal extraction protocol for *Yersinia* intracellular metabolites was investigated. The effect of five extraction protocols consisting of different extraction solvent systems (60% methanol, 100% methanol, acetonitrile/methanol/water (2:2:1), chloroform/methanol/water (2:1:1), and 60% ethanol) on *Yersinia* metabolic profiles were compared. The number of detected peaks, sample-to-sample variation, and metabolite yield were used as criteria. Extracted metabolites were analyzed by ^1^H-NMR and principal component analysis (PCA), as well as partial least squares discriminant analysis (PLS-DA) multivariate statistics. The extraction protocol using 100% methanol as the extraction solvent provided the highest number of detected peaks for both *Yersinia* species analyzed, yielding more spectral information. Together with the reproducibility and spectrum quality, 100% methanol extraction was suitable for intracellular metabolite extraction from both species. However, depending on the metabolites of interest, other solvents might be more suitable for future studies, as distinct profiles were observed amongst the extraction methods.

## 1. Introduction

The metabolome of any organism is complex, containing thousands of chemically and physically diverse metabolites [1,2]. The goal of untargeted metabolomics is to obtain as much information about the metabolome as possible using efficient, reproducible, and relatively affordable methods. Comparison and optimization of methods at the sample preparation, analysis, and interpretation levels are crucial [3]. Moreover, metabolomics methods should be optimized for each type of sample organism of interest [4,5,6].

One-dimensional (1D) ^1^H-NMR is an unbiased, nonselective, and nondestructive approach to characterizing the metabolome, and data can be obtained in a high-throughput manner [7]. The spectrum of a 1D ^1^H-NMR experiment contains a complex mixture of proton signals plotted along a chemical shift value (ppm) which describes the chemical environment and structural characteristics of specific metabolites. Furthermore, the intensity values of each proton signal are directly proportional to the concentration of the corresponding metabolite. 1D ^1^H-NMR coupled with multivariate statistics in metabolomics is commonly employed for the global analysis of the metabolome [8,9].

Statistical analysis of 1D ^1^H-NMR spectrum often utilizes principal component analysis (PCA) and partial least squares discriminant analysis (PLS-DA) [10,11]. These methods allow evaluation of clustering patterns between samples. A spectrum is often divided into numerous variables, called bins, which describe the spectrum [12]. PCA and PLS-DA reduces this information to a single point on a 2D or 3D score plot, based on the influence of particular variables in which maximum variance between samples is attributed. These clustering methods allow simple evaluation of sample-to-sample variation and, thus, the interpretation of reproducibility and class differences [13]. Experimental parameters should be identical amongst samples to obtain reliable data [7].

The genus *Yersinia* belongs to the family of *Enterobacteriaceae* and is composed of three human-pathogenic species: *Yersinia pestis*, the causative agent of plague, *Y. enterocolitica*, and *Y. pseudotuberculosis* [14]. The two *Yersinia* species, *Y. enterocolitica* and *Y. pseudotuberculosis*, that are enteropathogenic for humans, are distributed worldwide, and frequently cause diarrhea in inhabitants of temperate and cold countries [15,16]. *Y. enterocolitica* is a major cause of foodborne disease resulting from consumption of contaminated food products, and leads to substantial economic cost [17]. Generally, *Yersinia* can survive in most natural environments or food matrices using highly adaptable metabolic pathways that are typical of free-living enterobacteria [18]. Improving the metabolic characterization of bacterial isolates is of key interest in identification, treatment, and pathogenesis studies.

Several NMR-based strategies have been proposed for bacterial characterization under different physiological conditions [19,20]. In the present study, metabolome sampling protocols with varying extraction solvents were evaluated by analyzing intracellular metabolites of representative enteropathogenic *Yersinia* using NMR spectroscopy. Such insights will give us information on which extraction solvents are most suitable for the global characterization of *Yersinia* metabolome by ^1^H-NMR.

## 2. Materials and Methods

### 2.1. Bacterial Strains and Culture Conditions

*Y. pseudotuberculosis* (NR-804) and *Y. enterocolitica* (NR-214) were generous gifts from the Biodefense and Emerging Infections Research Resources Repository (BEI Resources; Manassas, VA, USA). Frozen stocks of *Y. enterocolitica* and *Y. pseudotuberculosis* were prepared cultured in tryptic soy broth (TSB; Sigma-Aldrich, St. Louis, MO, USA) and incubated at 30 °C for 24 h. Fresh cultures were used for propagation in 1600 mL total culture volume at 1:10 dilution. Inoculated cultures were grown at 30 °C, shaking for 24 h. Initial and final optical density measurements at 600 nm were recorded on a Power Wave XS O.D. Reader (BioTek, Winooski, VT, USA).

### 2.2. Sampling and Metabolite Extraction

After incubation, cultures were homogenized, divided into aliquots of 50 mL, and submerged in ice to inhibit cellular metabolism. Cell harvesting was performed by centrifugation of the aliquots at 3219*g* for 10 min at 4 °C. The supernatant was discarded, and the pellets were washed with an equal volume (25 mL) of 1× phosphate-buffered saline (PBS) followed by the same centrifugation procedure (3219*g* for 10 min at 4 °C). The resulting pellets were processed to extract the comprehensive metabolites using different extraction solvents: 60% methanol, 100% or pure methanol (PM), 60% ethanol (ETH), acetonitrile/methanol/water (AMW; 2:2:1), and chloroform/methanol/water (CMW; 2:1:1). Briefly, the washed pellet was suspended in 1 mL of the extraction solvent precooled to −20 °C. The suspension was vortexed for 30 s and sonicated for 15 s twice. The sonicated suspension was then centrifuged at 17,949*g* for 10 min at room temperature to remove cellular debris. The supernatant (1 mL total) was collected and dried using a vacuum centrifuge overnight at 30 °C. For CMW extracts, the polar fraction of the supernatant and the sample was re-extracted with 500 µL of 50% methanol by vortexing for 30 s. The dry extracts were then stored at −80 °C until NMR analysis.

### 2.3. NMR Data Acquisition

Before analysis, extracts were reconstituted in 700 µL of deuterium oxide (D_2_O; Sigma-Aldrich) containing 0.05% (w/w) trimethylsilylpropanoic acid (TSP), centrifuged (17,949*g*, 10 min, room temperature), and 650 µL of supernatant was transferred to an NMR tube (Sigma-Aldrich). Six replicates were analyzed per extraction method.

One-dimensional high-resolution ^1^H-NMR spectra were acquired on a Bruker Avance II 400 MHz spectrometer (Bruker Analytik, Rheinstetten, Germany). All data were collected at a temperature of 300 K. A standard one-dimensional proton NMR experiment with water presaturation (^1^H ZGPR) was performed for each sample. Experiments were run with 16 dummy scans, 128 acquisition scans, and a relaxation delay of 2 s. The spectral width was 16 ppm, and 32 K data points were collected. All free induction decays (FIDs) were subjected to an exponential line-broadening of 0.3 Hz. Upon Fourier transformation, each spectrum was manually phased, automatically baseline corrected, and referenced to the internal standard TSP at 0.0 ppm using TopSpin v3.5 software (Bruker Analytik).

### 2.4. Pre-Processing

The residual H_2_O NMR resonance between 4.6 and 5.0 ppm was excluded from analyses. Spectra were binned from 0.5 to 10 ppm. Each spectrum was referenced by setting the TSP peak to 0.0 ppm. A table of the average sum of integral values for bins with a width of 0.025 ppm, using the MestRe Nova v11.0.2 (Santiago de Compostela, Spain) binning scheme, was then exported to Microsoft Excel. All spectra tables were combined into a single file, then imported into MetaboAnalyst 3.0 tool suite for multivariate analysis [21]. All data were pareto-scaled and normalized to the sum of each spectrum.

Compound identification was done using the Chenomx NMR Suite v8.31 (Edmonton, Alberta, Canada), existing literature, and publicly available compound databases (Human Metabolome Database, HMDB [22]; Madison Metabolomics Consortium Database, MMCD [23]). A list of identified compounds for each extraction method can be found in the Supporting Materials (Table S1). Quantification of identified compounds were done by manual integration of corresponding peaks or peak clusters and importing the integral dataset into MetaboAnalyst 3.0 (Table S2). Metabolite levels were then normalized to the TSP peak. Also, the Kyoto Encyclopedia of Genes and Genomes (KEGG) [24] database for was used for pathway analyses.

## 3. Results and Discussion

**Figure 1:**
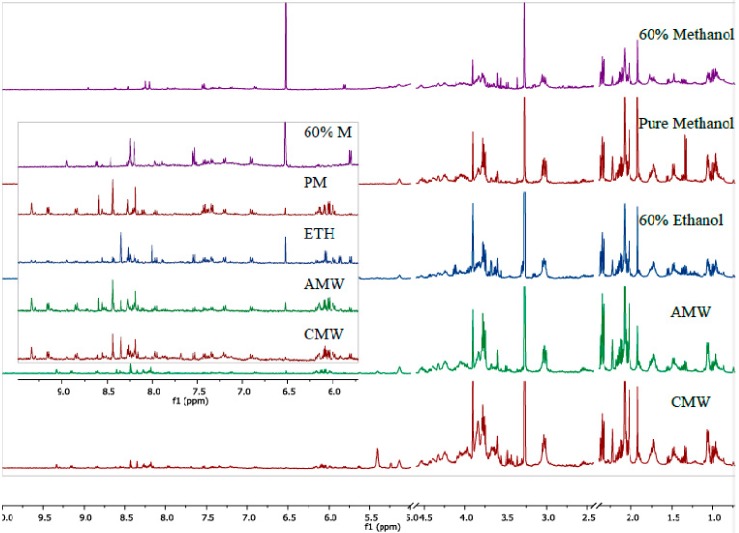
Representative ^1^H-NMR spectra of the polar fraction of the metabolome of *Yersinia enterocolitica* extracted using the tested extraction methods: 60% methanol, pure methanol (PM), 60% ethanol (ETH), acetonitrile/methanol/water (AMW; 2:2:1), and chloroform/methanol/water (CMW; 2:1:1).

**Figure 2:**
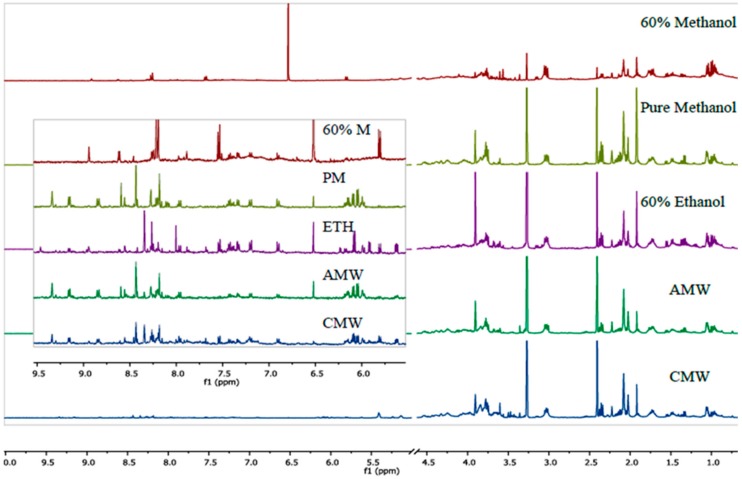
Representative ^1^H-NMR spectra of the polar fraction of the metabolome of *Y. pseudotuberculosis* extracted using the tested extraction methods: 60% methanol, PM, 60% ETH, AMW; 2:2:1, and CMW; 2:1:1.

For this experiment, we analyzed the quality of the spectra, variation among samples using PCA analyses, overall metabolite yield, and the number of metabolites. Comparison of representative spectra for *Y. enterocolitica (*Figure 1) and *Y. pseudotuberculosis* (Figure 2) showed that the quality of spectra between sample classes are similar in terms of resolution. Distinct profiles were observed quantitatively, especially from the downfield region of the spectra (4.9–9.5 ppm).

**Figure 3:**
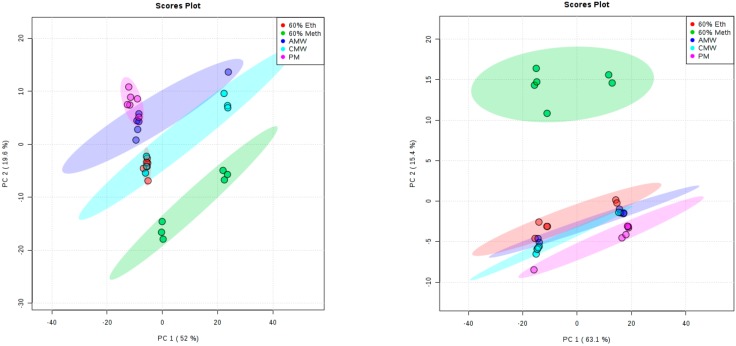
PCA scores plots of the polar fraction of the metabolome of (**left**) *Y. enterocolitica* and (**right**) *Y. pseudotuberculosis* (3D score plot). Different groups represent homogenates extracted using the following methods: 60% methanol, PM, 60% ETH, AMW; 2:2:1, and CMW; 2:1:1. For each extraction method, 95% confidence intervals (two standard deviations around the mean, represented by ovals) are shown.

Principal component analysis was used to compare the sample-to-sample variation of metabolic fingerprints derived from six replicates per method for both *Y. enterocolitica* and *Y. pseudotuberculosis*. From the PCA scores plot (Figure 3), *Y. enterocolitica* extraction methods showed separation along principal component 1 (PC1), which accounted for 52% of total variation. PC2 accounted for 19.6% and can be attributed to the variation within groups, particularly for the 60% methanol and chloroform/methanol/water (2:1:1) samples. Extraction methods using 60% ethanol, 100% methanol, and acetonitrile/methanol/water (2:2:1) showed great reproducibility. For Y. *pseudotuberculosis* samples, the score plot represented the clustering of the samples in Figure 3. The result was not so similar to *Y. enterocolitica* samples—the majority of the replicates showed good reproducibility except for 1 or 2 extreme samples that deviate from other replicates, as evidenced by the lack of a tight cluster formation in the PCA score plot. The CMW samples show significant clustering with one deviating replicate; the same was observed for the 100% methanol samples.

**Figure 4:**
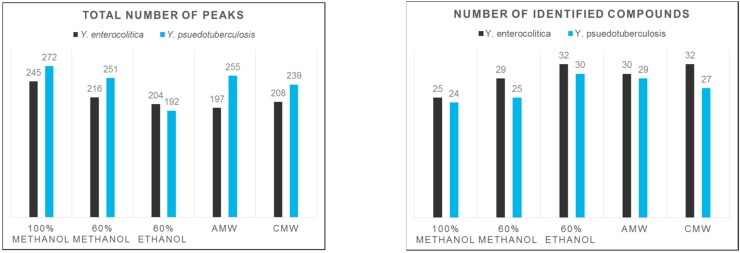
Comparison of the total number of peaks (**left**) and number of identified compounds (**right**) for each representative spectrum corresponding to the solvent treatment.

Comparison of the total number of metabolite-derived peaks can provide clues as to which extraction solvent gives the maximum number of metabolites. Figure 4 shows a comparison of the total number of detected peaks for each extraction method. For *Y. enterocolitica* and *Y. pseudotuberculosis*, 100% methanol extraction yielded the most ^1^H-NMR signals.

**Figure 5:**
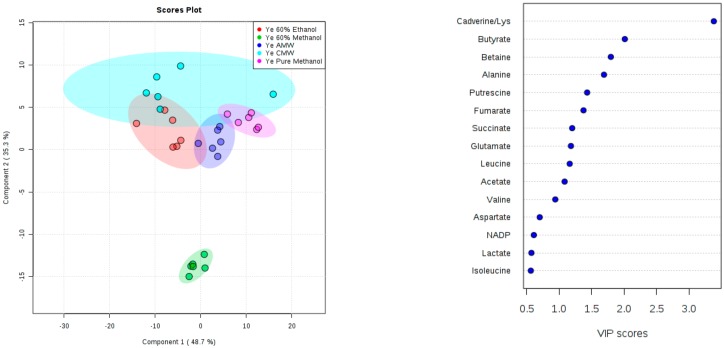
Partial least squares discriminant analysis (PLS-DA) score plot (**left**) and variable importance in projection (VIP) score plot derived from PLS-DA analysis (**right**) based on 36 identified compound peak areas of the polar fraction of the metabolome of *Y. enterocolitica* for different extraction methods: 60% methanol, PM, 60% ETH, AMW; 2:2:1, and CMW; 2:1:1. Lys: lysine.

**Figure 6:**
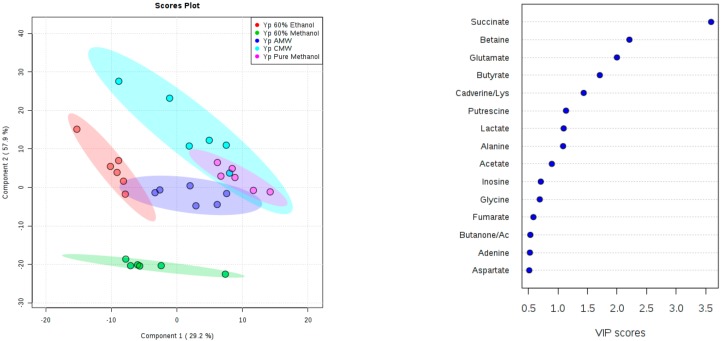
PLS-DA score plot (**left**) and VIP score plot derived from PLS-DA analysis (**right**) based on 36 identified compound peak areas of the polar fraction of the metabolome of *Y. pseudotuberculosis* of the different extraction methods: 60% methanol, PM, 60% ETH, AMW; 2:2:1, and CMW; 2:1:1. Ac: acetoin.

Additionally, we identified compounds using the Chenomx NMR Suite, which contains a compound database of 197 compounds for 400 MHz experiments. Also shown in Figure 4 is a comparison of the number of identified compounds for each extraction method. An integral region was determined for the peak(s) corresponding to a particular compound (Table S3). The compound areas or concentrations were then subjected to multivariate statistics, to assess the reproducibility and compound variations among the extraction methods. For both species of *Yersinia*, in the supplementary document (S4), PCA score plots showed that CMW solvent extractions have the least reproducible compound concentrations, as indicated by the larger shaded region (95% confidence region). For *Y. enterocolitica*, 60% methanol, AMW, and pure methanol solvents showed greater reproducibility of compound concentrations as seen in the PCA and PLS-DA score plot (Figure 5). In Figure 6, the PLS-DA score plot showed pure methanol as the most reproducible compound concentrations for *Y. pseudotuberculosis*. Variable importance in projection (VIP) plots derived from PLS-DA analysis, also shown in Figure 5 and Figure 6, showed the top compounds scored based on their contribution to the separation of extraction methods, as seen on the PLS-DA score plots.

**Figure 7:**
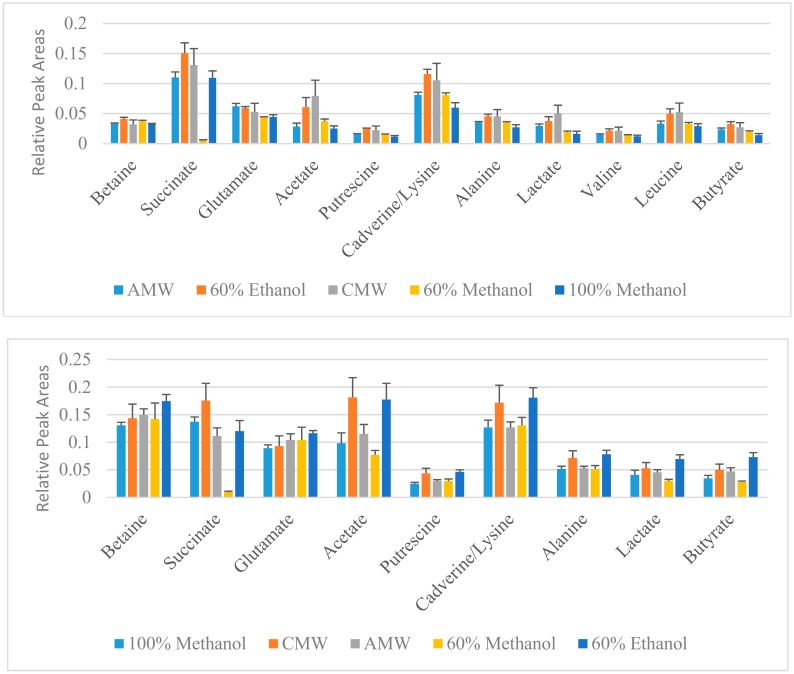
Relative metabolite levels extracted from *Y. enterocolitica* cultures (**top**) and *Y. pseudotuberculosis* (**bottom**).

Compounds that significantly contribute (VIP >1) to differences observed between the extraction methods in the score plots are shown in Figure 7. For *Yersinia enterocolitica*, 11 metabolites have VIP scores greater than 1, indicating these metabolites are the most influenced based on the evaluated extraction solvents used during the extraction of intracellular metabolites. These metabolites include amino acids (leucine, valine, alanine, lysine, and glutamate), polyamines (cadverine and putrescine), betaine, and other organic acids including succinate, acetate, and butyrate. Generally, higher levels of these compounds are extracted using the 60% ethanol or CMW solvent, with CMW being the least reproducible given the relatively larger standard error bars shown in Figure 7. This high degree of variation within the CMW method is also reflected in the PLS score plot in Figure 6. For *Yersinia pseudotuberculosis*, nine metabolites have VIP scores greater than 1, and their relative levels are also shown in Figure 7. Similar to *Y. enterocolitica*, 60% ethanol or CMW methods extracted slightly higher amounts of the major metabolites. 

**Figure 8:**
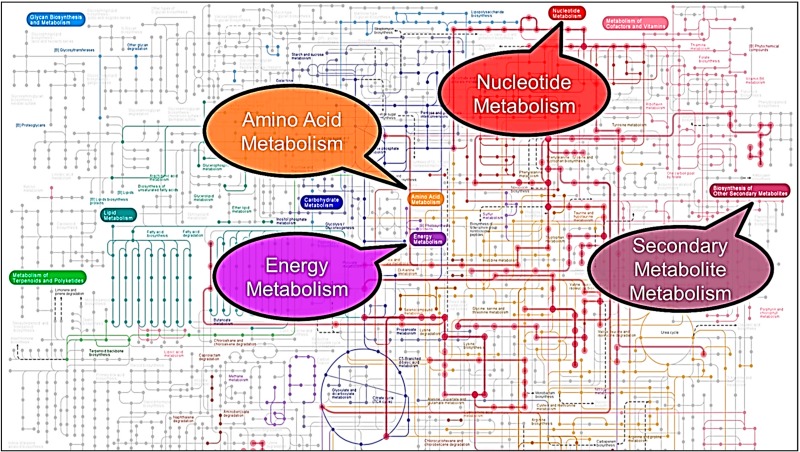
Overview of KEGG-based metabolic network derived from all detectable compounds extracted from *Yersinia.*

Figure 8 shows a general metabolic network for *Yesinia* species generated from the KEGG database. Pathways highlighted in red indicate the association of extracted metabolites with particular pathways. While the significance of particular pathways is based on the compounds detected for each extraction method, the evaluated extraction methods provided a means to evaluate metabolic changes in amino acid, energy, nucleotide, and secondary metabolite metabolisms. Pathway elucidation, from a metabolomics standpoint, depends on the detection of compounds relating to particular metabolic pathways. Identification of perturbed metabolic pathways of stressed cells is crucial across a number of areas, including drug discovery and pathology [25]. Based on the metabolic profile for each extraction method, the compounds identified were subjected to pathway analysis utilizing the KEGG pathway database. Differences in the compound profile for each method yielded different metabolic pathways that could be monitored. The pathway analysis revealed a number of pathways that could be monitored based on the metabolic profile for each extraction method (Figure 8).

## 4. Conclusions

Based on the number of detected peaks, sample-to-sample variation, and the ease and speed of the experimental efficiency, 100% methanol was the better of the five extraction solvents tested for the extraction of intracellular metabolites from *Y. enterocolitica* and *Y. pseudotuberculosis*, in terms of global fingerprinting. For more targeted assays, the choice of extraction solvent is highly dependent on the reproducibility and the identification of specific metabolites as different solvents may readily extract different metabolites, thus allowing evaluation of relevant metabolic pathways. In this case, based on the identified compounds, the CMW solvents for both species resulted in the most variation amongst replicates in compound concentrations based on the large 95% confidence limits, as seen in Figure 5. Due to the number of amino acids identified, amino acid metabolism can easily be monitored for all extraction methods in both species, in addition to other major metabolisms indicated in Figure 8.

No single platform can reliably measure the chemically and structurally diverse metabolites of the metabolome, especially for untargeted metabolites [26,27]. Multiplatform approaches are used to expand coverage of the metabolome [28,29]. While NMR is praised for its high technical reproducibility, minimal sample preparation, and rapid analysis [30], other platforms, such as liquid chromatography-mass spectrometry (LC-MS) offer much higher sensitivity and molecular specificity [27,30]. With the incorporation of other analytical platforms, we can extend metabolome coverage and, therefore, expand baseline metabolomics data for future studies with *Yersinia*.

## Supplementary Materials

The following are available online at http://www.mdpi.com/2409-9279/1/4/45/s1, Table S1: list of compounds present or absent in extraction, Table S2: dataset of peak areas corresponding to identified metabolites, Table S3: chemical shift regions for peak corresponding to identified metabolites. Figures S4: PCA score plots comparing reproducibility of extraction methods for both species. Additional supplementary data includes stacked spectra, original data location/directory and MetaboAnalyst 3.0 pathway analyses derived from compound list obtained from each extraction method.
