# Supplementary material for: Influence of Metabolite Extraction Methods on 1H-NMR-Based Metabolomic Profiling of Enteropathogenic Yersinia
Source: Methods Protoc. 2018 Nov 20;1(4):45. doi: 10.3390/mps1040045 (PMC6481057; doi:10.3390/mps1040045)
Supplement: Supplementary file 1 [file mps-01-00045-s001.zip › Supplemental Info/Compound list (Table S1).docx]

Compound list identified via Chenomx for Y. enterocolitica (A) and Y. pseudotuberculosis (B). 1 = present, 0 = not present.

A.

| **Metabolite** | **60% Methanol** | **Pure Methanol** | **AMW** | **60% Ethanol** | **CMW** | **ppm** |
| --- | --- | --- | --- | --- | --- | --- |
| 1,3 Dihydroxyacetone | 1 | 1 | 1 | 1 | 1 | 3.604 |
| 3-Hydroxyisobutyrate | 1 | 1 | 1 | 1 | 1 | 1.07 |
| Acetate | 1 | 1 | 1 | 1 | 1 | 1.92 |
| Adenine | 1 | 1 | 1 | 1 | 0 | 8.222 |
| ADP | 0 | 0 | 1 | 0 | 0 | 8.516 |
| Alanine | 1 | 1 | 1 | 1 | 1 | 1.483 |
| AMP | 0 | 1 | 1 | 1 | 1 | 8.598 |
| Aspartate | 0 | 0 | 0 | 1 | 1 | 2.801 |
| ATP | 0 | 0 | 1 | 1 | 0 | 8.516 |
| Betaine | 1 | 1 | 1 | 1 | 1 | 3.27 |
| Butanone/Acetoin | 1 | 1 | 1 | 1 | 1 | 2.225 |
| Butyrate | 1 | 1 | 1 | 1 | 1 | 0.885 |
| Cadverine | 1 | 1 | 1 | 1 | 1 | 1.723 |
| Fumarate | 1 | 1 | 1 | 1 | 1 | 6.52 |
| Glucose | 1 | 0 | 1 | 1 | 1 | 5.237 |
| Glutamate | 1 | 1 | 1 | 1 | 1 | 2.358 |
| Glycine | 1 | 1 | 1 | 1 | 1 | 3.561 |
| Hypoxanthine | 1 | 0 | 0 | 1 | 0 | 8.2 |
| IMP | 1 | 1 | 1 | 1 | 1 | 8.559 |
| Inosine | 1 | 0 | 1 | 1 | 1 | 8.346 |
| Isoleucine | 1 | 1 | 1 | 1 | 1 | 1.026 |
| Lactate | 1 | 1 | 1 | 1 | 1 | 1.33 |
| Leucine | 1 | 1 | 1 | 1 | 1 | 0.967 |
| Lysine | 1 | 1 | 1 | 1 | 1 | 1.723 |
| Maltose | 1 | 0 | 1 | 1 | 1 | 5.409 |
| Methionine | 1 | 0 | 0 | 1 | 1 | 2.146 |
| NADP+ | 1 | 1 | 1 | 1 | 1 | 8.84 |
| Niacinamide | 0 | 0 | 0 | 0 | 1 | 8.94 |
| Nicotinate | 0 | 0 | 0 | 0 | 1 | 8.94 |
| Phe | 1 | 1 | 1 | 1 | 1 | 7.334 |
| Putrescine | 1 | 1 | 1 | 1 | 1 | 1.772 |
| Succinate | 0 | 1 | 1 | 1 | 1 | 2.41 |
| Tyrosine | 1 | 1 | 1 | 1 | 1 | 6.903 |
| UDP-glucoronate | 1 | 0 | 0 | 0 | 1 | 7.962 |
| Uracil | 1 | 1 | 1 | 1 | 1 | 7.537 |
| Valine | 1 | 1 | 1 | 1 | 1 | 0.997 |
| **Total Compounds** | **29** | **25** | **30** | **32** | **32** |  |

B.

| **Metabolite** | **60% Methanol** | **60% Ethanol** | **Pure Methanol** | **CMW** | **AMW** | **ppm** |
| --- | --- | --- | --- | --- | --- | --- |
| 1,7 Dimethylxanthine | 1 | 1 | 0 | 0 | 0 | 3.32 |
| 4-pyroxidate | 0 | 1 | 0 | 0 | 1 | 2.454 |
| Adenine | 1 | 1 | 1 | 1 | 1 | 8.222 |
| Alanine | 1 | 1 | 1 | 1 | 1 | 1.483 |
| AMP | 0 | 1 | 0 | 1 | 1 | 8.598 |
| Aspartate | 1 | 1 | 0 | 1 | 1 | 2.801 |
| ATP | 1 | 1 | 0 | 1 | 1 | 8.516 |
| Betaine | 1 | 1 | 1 | 1 | 1 | 3.27 |
| Cadverine | 1 | 1 | 1 | 1 | 1 | 1.723 |
| Fumarate | 1 | 1 | 1 | 0 | 0 | 6.52 |
| Glucose | 0 | 1 | 1 | 1 | 1 | 5.237 |
| Glutamate | 1 | 1 | 1 | 1 | 1 | 2.358 |
| Glycine | 1 | 1 | 1 | 1 | 1 | 3.561 |
| Hypoxanthine | 1 | 1 | 0 | 1 | 1 | 8.2 |
| IMP | 1 | 1 | 1 | 1 | 1 | 8.559 |
| Inosine | 0 | 1 | 1 | 1 | 1 | 8.346 |
| Isoleucine | 1 | 1 | 1 | 1 | 1 | 1.026 |
| Lactate | 1 | 1 | 1 | 1 | 1 | 1.33 |
| Leucine | 1 | 1 | 1 | 1 | 1 | 0.967 |
| Lysine | 1 | 1 | 1 | 1 | 1 | 1.723 |
| Maleate | 1 | 1 | 1 | 1 | 1 | 6.041 |
| Methionine | 1 | 1 | 1 | 1 | 1 | 2.146 |
| NADP+ | 1 | 1 | 1 | 1 | 1 | 8.84 |
| Niacinamide | 1 | 1 | 0 | 1 | 1 | 8.94 |
| Phe | 1 | 1 | 1 | 1 | 1 | 7.334 |
| Putrescine | 1 | 1 | 1 | 1 | 1 | 1.772 |
| Succinate | 0 | 1 | 1 | 1 | 1 | 2.41 |
| Tyrosine | 1 | 1 | 1 | 1 | 1 | 6.903 |
| UMP | 0 | 0 | 1 | 0 | 1 | 8.1 |
| Uracil | 1 | 1 | 1 | 1 | 1 | 7.537 |
| Valine | 1 | 1 | 1 | 1 | 1 | 0.997 |
| **Total Compounds** | **25** | **30** | **24** | **27** | **29** |  |
