# Supplementary material for: Influence of Metabolite Extraction Methods on 1H-NMR-Based Metabolomic Profiling of Enteropathogenic Yersinia
Source: Methods Protoc. 2018 Nov 20;1(4):45. doi: 10.3390/mps1040045 (PMC6481057; doi:10.3390/mps1040045)
Supplement: Supplementary file 1 [file mps-01-00045-s001.zip › Supplemental Info/PCA Score Plots (S4).docx]

PCA score plots for *Y. enterocolitica* and *Y. pseudotuberculosis* extraction – Supplemental Information


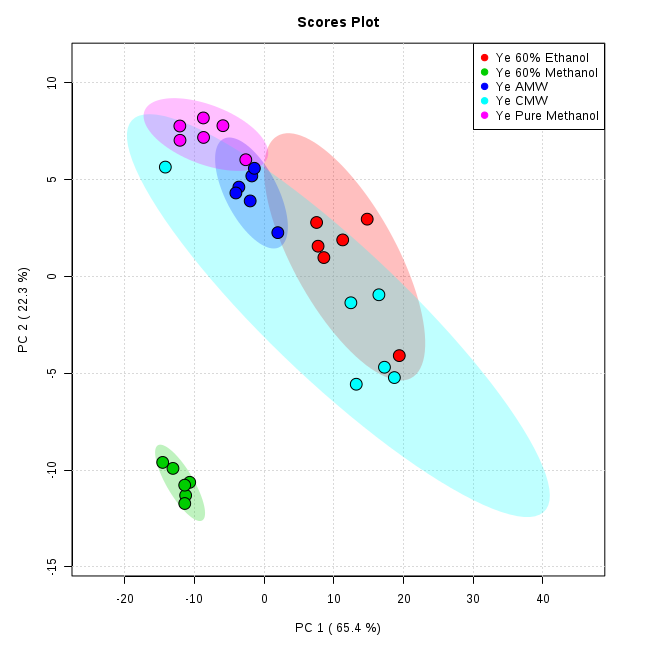


PCA score plot based on 36 identified compound peak areas of the polar fraction of the metabolome of *Y. enterocolitica* for different extraction methods: 60% methanol, pure methanol (PM), 60% ethanol (ETH), acetonitrile:methanol:water (AMW; 2:2:1), and chloroform:methanol:water (CMW; 2:1:1).


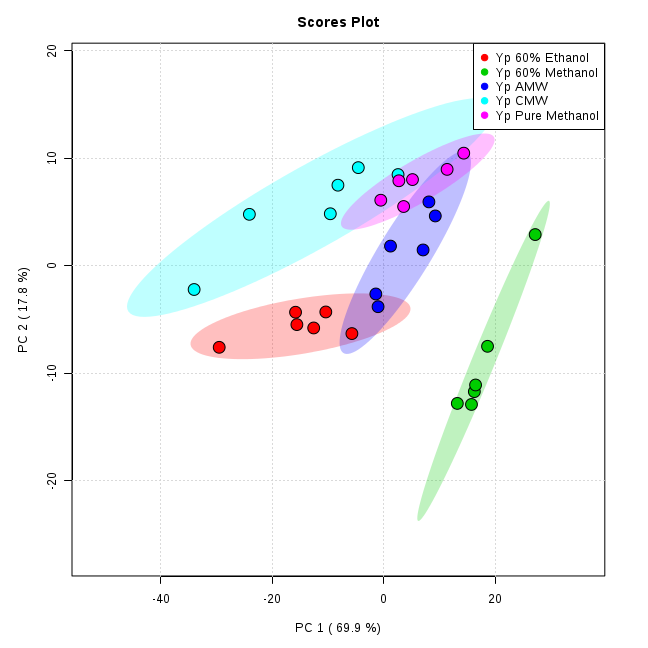


PCA score plot based on 36 identified compound peak areas of the polar fraction of the metabolome of *Y. pseudotuberculosis* of the different extraction methods: 60% methanol, pure methanol (PM), 60% ethanol (ETH), acetonitrile:methanol:water (AMW; 2:2:1), and chloroform:methanol:water (CMW; 2:1:1).
